# Longitudinal Relationship between Bullying Victimization and Non-Suicidal Self-Injury among Chinese Adolescents: The Buffering Roles of Gratitude and Parental Autonomy Support

**DOI:** 10.3390/ijerph20021440

**Published:** 2023-01-12

**Authors:** Nini Wu, Jianhong Mo, Anluan Wen, Haoer Ou, Weixin Gu, Yunqing Qiu, Lixin Yuan, Xiaoyu Lan

**Affiliations:** 1 Department of Psychology, School of Education, Guangdong University of Education, Guangzhou 510310, China; 2 Department of Psychology, The Chinese University of Hong Kong, Hong Kong, China; 3 Promenta Research Center, Department of Psychology, University of Oslo, 0373 Oslo, Norway

**Keywords:** adolescents, bullying victimization, non-suicidal self-injury, gratitude, parental autonomy support

## Abstract

Drawing on the resilience-oriented socioecological framework, the current study contributes to scarce scholarship by exploring intrapersonal (i.e., gratitude) and interpersonal (i.e., parental autonomy support) factors in the longitudinal association between bullying victimization and adolescent non-suicidal self-injury (NSSI). Participants were 238 Chinese adolescents (*M*age at Time 1 (T1) = 13.45 years; 106 girls and 132 boys) based on a two-wave prospective design with data spanning one year. At T1, adolescents self-rated all study variables, and at Time 2 (T2), youth again reported their NSSI. The results showed a significant main effect (*b* = 0.12, *SE* = 0.05, *p* = 0.04), indicating that bullying victimization was positively related to T2 NSSI one year later, even controlling for T1 NSSI. Moderation analyses further indicated that parental autonomy support buffered against the positive association between bullying victimization and T2 NSSI, but only when adolescents experienced lower levels of gratitude. Specifically, for adolescents with lower levels of gratitude, high levels of parental autonomy support, in a compensatory way, prevented adolescents from NSSI after victimization occurred (*b* = −0.03, *SE* = 0.09, *p* = 0.78); by contrast, for those with higher levels of gratitude, bullying victimization was not significantly related to T2 NSSI, regardless of the levels of parental autonomy support (*b* = 0.07, *SE* = 0.04, *p* = 0.59 for higher parental autonomy support; *b* = 0.01, *SE* = 0.07, *p* = 0.93 for lower parental autonomy support). These findings suggest that gratitude and parental autonomy support, manifesting in a compensatory interaction pattern, could serve as targeted agents for breaking the vicious linkage between bullying victimization and NSSI.

## 1. Introduction

School bullying has been widely recognized as a serious public health issue with substantial negative consequences around the world. Bullying is commonly defined as aggressive behavior in which someone in a position of power intentionally and repeatedly causes another person’s injury or discomfort [1]. Bullying can take the forms of physical assault (e.g., pushing and hitting), verbal harassment (e.g., verbal taunting and rumor spreading), and isolation [2]. Bullying victimization refers to the experiences of being bullied by others, including physical victimization, relational victimization, and verbal victimization [3]. Bullying victimization generally peaks in early adolescence [4] and remains stable or decreases during adolescence [5], with a prevalence rate (being victimized at least once) of approximately 30.4% [6]. Adolescence spanning the age range of 12–18 [7] is traditionally considered to be the developmental period in life when peer influence is the most intense [8]. Being victimized by their peers has become one of the most critical risk factors for adolescent maladjustment [9]. A large number of empirical studies and meta-analyses have shown that peer victimization is more prevalent among youth who have engaged in non-suicidal self-injury (NSSI) compared to youth without such a history [10,11,12]. The majority of extant literature supporting such an association between bullying victimization and NSSI has nevertheless been dominated by cross-sectional studies [10,13]. The existing but limited longitudinal studies have unfortunately demonstrated inconsistent findings [14,15]. Therefore, a prospective longitudinal design is urgently needed to clarify this divergent evidence and further deepen the conceptual understanding of whether and when bullying victimization has a longitudinal relationship with NSSI. 

Moreover, given the potential risk of bullying victimization for NSSI, attention must be given to potential protective factors that mitigate such a vicious effect. In doing so, we integrated the socioecological theory [16] with a risk-protection perspective [17]. Drawing from two waves of youth-report data, the objectives of this research are to (a) examine the longitudinal relationship between bullying victimization and adolescent NSSI; (b) investigate whether gratitude, as an intrapersonal factor and parental autonomy support, as an interpersonal factor serve as buffering agents against adolescent NSSI when they experience victimization. Addressing these objectives can offer potential opportunities for interrupting cascading processes of bullying victimization on adolescent NSSI, which can inform targeted interventions aimed at decreasing NSSI.

### 1.1. Bullying Victimization and Adolescent Non-Suicidal Self-Injury

Non-suicidal self-injury (NSSI) refers to the direct, deliberate, and socially unacceptable destruction of body tissue without the intention to die [18]. Common NSSI behaviors include self-cutting, self-burning, punching, or hitting oneself [19]. The interpersonal model of NSSI has proposed that negative interpersonal events may induce stress or tension, which increases the risk for NSSI [20]. Adolescence is a developmental period undergoing increased risk for negative peer influence, probably due to substantial biological and psychosocial changes [21]. After being victimized, negative emotions can be evoked. For example, victims of school bullying are likely to doubt themselves and attribute their victimization to their own faults and weaknesses [22]. They also tend to ruminate over their past victimization experiences [23]. Under such circumstances, adolescents may experience increased negative emotions, such as shame, stress, embarrassment, frustration, fear, and depression [12,24,25,26]. All of these negative emotions may activate adolescents to engage in NSSI with the aim of releasing emotional pressure. 

Indeed, a large body of cross-sectional studies has demonstrated that bullying victimization is positively related to the engagement of NSSI [10,13]. However, findings from the limited longitudinal studies were conflicting. For example, Jiang et al. (2016) found that peer victimization predicted increased NSSI frequency among Chinese adolescents over a one-year period [14], whereas Heilbron and Prinstein (2010) found no longitudinally significant effects of peer victimization on adolescent NSSI engagement two years later [15]. These inconsistent findings highlight the need for additional empirical studies to elucidate the longitudinal association of bullying victimization with NSSI.

### 1.2. Gratitude and Parental Autonomy Support as Moderators

Our conceptualization of the moderating roles of gratitude and parental autonomy support is grounded in the socioecological theory [16] and risk-protection framework [17]. The socioecological theory assumes that individual development is shaped by the interplay between individuals themselves and multiple systems in which individuals are embedded [16]. This assumption is compatible with the risk-protection framework, another theoretical perspective guiding this study, which seeks to understand the factors that protect individuals from vulnerability in the face of adversity. Risk is defined as a condition within adolescents’ socialization contexts that potentially increase the likelihood of unfavorable development outcomes [27]; in the present research, the risk is conceptually symbolized by bullying victimization. Protective factors, by contrast, refer to the safeguards that shield adolescents from risk agents by reducing the likelihood of negative outcomes [27]. Given our primary interest in understanding how adolescents cope with bullying victimization and how significant others (e.g., parents) function as an interpersonal protective factor, we focused on positive individual attributes and parental-related support as possible protective factors; in this perspective, gratitude and parental autonomy support that adolescents may draw upon when they encounter victimization come into our attention. Specifically, scholars have identified numerous personal characteristics [14,28,29] and parental support [30,31] that serve as an ameliorative function in the bullying victimization context. Despite growing popularity in scholarly literature, insufficient attention has been paid to adolescent gratitude and parental autonomy support, given their potential to promote psychological resources through cultivating positive emotions and appraisal, acknowledging adolescents’ feelings, and allowing adolescents to make choices on their own. Socioecological theory and prior empirical studies have provided solid support for the interaction effect of gratitude and parental autonomy support on adolescent development [16,32]. However, their independent and combined moderating effects in the linkage between bullying victimization and adolescent NSSI have not been empirically tested. In closing those gaps, the independent moderating role of gratitude and parental autonomy support, as well as their joint moderating effects, are therefore explored in the present study.

#### 1.2.1. Gratitude

Since the 2000s, gratitude has attracted increasing scholarly attention within psychological research. Gratitude is commonly defined as the tendency to recognize other people’s kindness or support and respond with grateful emotions [33]. Gratitude usually occurs after people receive aid, which is perceived as costly, valuable, and altruistic [34]. To date, an increasing number of studies have identified a variety of beneficial effects of gratitude on physical health, mental health, and well-being [35,36]. For instance, grateful people tend to have more strong social relationships by showing more forgiveness, empathy, and warmth to others [37,38]. Being grateful for the things in life allow individuals to perceive less stress and experience greater life satisfaction [39,40]. Furthermore, individuals with high gratitude tend to report high levels of positive affect and low levels of negative feelings, including depression and anxiety [33].

The current study examined gratitude as a buffer against the linkage between bullying victimization and NSSI, regarding gratitude as a coping mechanism for emotional regulation to overcome the adverse effects of bullying victimization, guided by the broaden-and-build theory [41]. Specifically, the broaden-and-build theory posits that experiences of positive emotions broaden individuals’ momentary thought-action repertoires, which in turn serve to build enduring personal resources, including physical, intellectual, social, and psychological resources [41]. In line with this theory, gratitude may mitigate the effects of bullying victimization on NSSI based on the following reasons. First of all, rather than focusing exclusively on the adverse experience (i.e., victimization experiences), gratitude helps adolescents possess a worldview that is more focused on the appreciation of the good things in life, including personal qualities, skills, and resources [42]. As a result, they may be less prone to seeing themselves as incompetent and more willing to consider themselves as resources and to encourage themselves. These actions may lead to less anxiety when facing adverse life circumstances (e.g., bullying victimization) [43]. Second, gratitude also cultivates positive reappraisal to demanding situations from an adaptive perspective [44,45]. Cognitive reappraisal can change the emotional impact elicited by the situation [44]. It helps not only down-regulate adverse emotions but also up-regulate positive emotions [46]. This gives adolescents a reason to feel as if the situation is not that bad after bullying victimization, and therefore, they may not spend more time thinking about it, blaming themselves, or immersing in depression, greatly reducing the probability of NSSI. Third, grateful people have stronger social support, which are vital resources for victims looking for help [47,48]. Taken together, gratitude is regarded as a protective factor against negative experiences and psychological difficulties [49,50].

Indeed, there has been evidence supporting the role of gratitude in alleviating the impact of negative contextual factors on adolescents’ maladjustment or mental health [45,51]. For example, in a sample of 738 college students in the U.S., self-reported gratitude significantly mitigates the positive effects of negative life events on psychological stress [51]. Lo et al. (2017) found that gratitude served as a buffer such that when experiencing higher (vs. lower) levels of gratitude [45], the strength from dominant parenting to adolescent suicidal ideation was weaker. 

To the best of our knowledge, very few studies have investigated the buffering role of gratitude in bullying victimization. One exception is Rey et al. (2019)’s investigation [52], suggesting that grateful people have broad-ranging thoughts and actions, which allows them to get access to resources and helps them overcome difficulties, thereby decreasing suicide risks (including depression, suicide intent, and behavior) after encountering victimization. Although NSSI, depression, suicidal ideation, and suicidal behavior are all suicidal risks, there are subtle conceptual differences among them. To be specific, NSSI, which refers to the act of harming oneself without conscious suicidal intent, is distinct from depression, suicidal ideation, and suicidal behaviors [53,54]. Different aspects of suicide-related outcomes have very different prevalence rates, functions, clinical correlates, and outcomes [55], elucidating the necessity and feasibility to explore the buffering role of gratitude in the linkage between bullying victimization and NSSI. 

#### 1.2.2. Parental Autonomy Support 

Parental autonomy support refers to parents’ active support of their offspring’s capacity to be self-initiating and self-determined by taking the perspective of their offspring into account, providing choices, and exchanging opinions and ideas [56,57]. In this study, we focused on the possible protective role of perceived parental autonomy support based on the following considerations. According to self-determination theory, autonomy is one of the three basic psychological needs that contribute to psychosocial adjustment [56]. The satisfaction of psychological needs for autonomy is particularly crucial for adolescents who are in increased demand for autonomy [57]. Although adolescence is often viewed as a period of increased peer influence, parents remain to be prominent figures, and the significance of parental support endures during this period of life [31,58]. Extant research based on self-determination theory has indicated that greater autonomy support is associated with less stress incursion and more active coping with stressful events [57,59], whereas perception of lower autonomy support has been found to be related to adolescent maladjustment [60,61]. 

Although the positive function of parental autonomy support in adolescent adjustment has been exponentially established, the buffering role of parental autonomy support is relatively understudied regarding the association between bullying victimization and adolescent NSSI. Based on the stress-buffering hypothesis [62], perceiving support may reduce the perception of harm posed by a specific situation, thus preventing the stress appraisal response. Guided by this stress-buffering hypothesis, it is reasonable to speculate that perceived parental autonomy support may be an active protector in preventing victims from engaging in NSSI. Parental autonomy support provides a safe avenue to express worries and grant emotional reassurance [58]. Feeling of autonomy, warmth, and being respected may embrace adolescents when they perceive their parents’ acknowledgments of their feelings and provide choices after victimization. Such emotional support may be particularly salient when adolescents experience bullying. Newman et al. (2005) have asserted that bullying is a chronic stressor and social support is one of the coping resources on which victims can draw [63]. Moreover, with great autonomy support from parents, adolescents tend to develop better skills or traits (e.g., emotional regulation strategies and resilience) [64,65], which can help them respond to stress in a more constructive way.

No empirical study has to date directly tested the moderating role of autonomy-supportive parenting in the linkage between victimization and NSSI, but indirect evidence has indicated that the negative consequences occur among victims partly depending upon parental support. For example, prior research has found that higher (versus lower) levels of parental communication buffer against the positive influence of bullying victimization on internalizing problems [31]. In a sample of 785 adolescents, the linkage between victimization and depression is less pronounced in adolescents who experienced high parental support [30].

### 1.3. The Current Study

To the best of our knowledge, research on the possible protective roles of gratitude and parental autonomy support linking bullying victimization and NSSI has not been empirically assessed. The present study aims to close those gaps and contribute to the scientific literature by testing whether and when gratitude and parental autonomy support buffered the linkage between bullying victimization and NSSI over time. The first research goal was to clarify the divergent evidence in the extant research by reconfirming the longitudinal relationship between bullying victimization and T2 NSSI over time after adjusting for T1 NSSI. The current study expected bullying victimization to be positively related to T2 NSSI after controlling for T1 NSSI (main effect). The second research goal was to test whether gratitude and/or parental autonomy support could act as positive resources that ameliorate NSSI caused by bullying victimization. Building on previous theoretical and empirical findings, we expected that the positive linkage between bullying victimization and NSSI would be weaker when adolescents reported higher (versus lower) levels of gratitude *or* parental autonomy support (two-way interaction). Additionally, we expected joint moderating effects of gratitude and parental autonomy support on the studied association. Specifically, this positive association between bullying victimization and NSSI would be the weakest for adolescent victims who simultaneously reported higher (versus lower) levels of gratitude and parental autonomy support (three-way interaction). Of important note, the research hypotheses regarding this three-way interaction involved a mixture of confirmatory and exploratory approaches. The expected three-way interaction was firmly informed by the overarching socioecological framework, whereas the direction of this three-way interaction was mainly exploratory due to the scarcity of literature concerning those complex interaction patterns.

## 2. Method

### 2.1. Participants

The current study was based on a sample of 238 adolescents (106 girls and 132 boys) from a larger longitudinal study on adolescent adjustment. At Wave 1, adolescents were between 12 and 15 years old (*M*age = 13.44 years, *SD* = 0.53). The participants were recruited from a public school in Foshan city, one of the most economically developed cities in South China. The 2021 annual gross domestic product in Foshan was 1215.7 billion RMB (Foshan Bureau of Statistics, 2021 [66]), ranking 3rd of the 21 cities in Guangdong province, China. At Wave 1, a total of 299 youth participated. The final sample for the current study included 238 students with both Wave 1 and Wave 2 data. The attrition rate was 20.4% due primarily to the following three reasons: (1) students did not provide their ID numbers, and in this regard, we were not able to match their data in two waves; (2) some of the students withdrew; (3) some of the students transferred schools. An attrition analysis was conducted to compare the 238 adolescents who had data at both waves with the 299 adolescents who did not have matched data at Wave 2 on all study variables. No significant difference between the two samples was found, except that the students who took part in both waves showed greater gratitude than those who participated in Wave 1 only. 

The median father and mother’s education level was high school completion. Among the 229 students providing valid data for father’s educational level, 0.4% did not complete primary school; 6.1% completed primary school; 36.2% completed middle school; 36.7% completed high school; 10.0% attained an associate degree; 10.0% attained a bachelor’s degree; and 0.4% attained a master’s degree; Among the 230 providing valid data for mother’s educational level, 1.7% did not complete primary school; 9.6% completed primary school; 43.5% completed middle school; 23.0% completed high school; 13.9% attained an associate degree; and 8.3% attained a bachelor’s degree. Among the 236 providing valid data on their living situation, 93.2% of students lived with their biological fathers, and 97.5% of students lived with their biological mothers. 

### 2.2. Procedure

The study was approved by the Institutional Review Board of the principal investigator’s university. Students were informed about the confidentiality of the data collected. They were also asked to provide written consent if they were willing to participate. During school hours, questionnaires were administrated by the trained school psychologist. Students were asked to fill in the questionnaires in the classroom independently. Approximately one year later (T2), students completed the questionnaires again. 

### 2.3. Measures

#### 2.3.1. Bullying Victimization 

At T1, bullying victimization was assessed with five items using the Bullying Perpetration/Victimization scale [67]. One sample item is: “How often has someone hit you in school or outside school.” Response options included the following: 1 = never, 2 = happened a year ago, 3 = a few times within a year, 4 = a few times a month, 5 = once a week, 6 = a few times a week, and 7 = at least once a day. A composite score was computed by averaging scores of the five items, with higher scores indicating higher levels of bullying victimization. The scale has demonstrated good reliability and validity among Chinese adolescents [12]. In the current sample, Cronbach’s alpha was 0.65. 

#### 2.3.2. Gratitude

At T1, gratitude was assessed using the six-item Gratitude Questionnaire [33]. One sample item is: “If I had to list everything that I felt grateful for, it would be a very long list.” Participants rated each item on a 5-point scale, ranging from 1 (not like me at all) to 5 (like me very much). After the two reverse-coded items were rescaled, a composite score was computed by averaging the scores of the six items, with higher scores indicating higher levels of gratitude. The questionnaire has demonstrated good reliability and validity among Chinese adolescents [68,69]. In the present study, Cronbach’s alpha was 0.72.

#### 2.3.3. Parental Autonomy Support

Following prior research, parental autonomy support at T1 was measured by the parental autonomy support questionnaire developed by Wang et al. (2007) [70]. This questionnaire consisted of two dimensions: choice-making and opinion exchanges. Sample items are, “My parents allow me to make choices whenever possible (choice-making)”; “My parents encourage me to give my ideas and opinions when it comes to decisions (opinion exchanges)”. Participants rated all items on a 5-point Likert-type scale ranging from 1 (completely disagree) to 5 (completely agree). A composite score was computed by averaging the scores of eight items, with higher scores indicating higher levels of perception of autonomy support from parents. Prior research has demonstrated good internal consistency of this scale in Chinese adolescents [57,71]. In this study, Cronbach’s alpha was 0.92.

#### 2.3.4. Non-Suicidal Self-Injury (NSSI)

At T1 and T2, participants were asked to report the frequency of the ways (e.g., self-cutting, carving, burning, etc.) in which they deliberately harmed themselves without the intention to die in the past years. These items were rated on a 7-point scale ranging from 1 (never) to 7 (at least once every day). A composite score for NSSI was computed by averaging the scores of the eleven items, with higher numbers indicating greater NSSI. Prior research has exhibited good psychometric properties of this scale among Chinese adolescents [24]; in the current sample, the internal consistency was also good in both time points (*α* = 0.91 at T1, *α* = 0.92 at T2). 

### 2.4. Analysis Plan

Data analyses were conducted in three steps. First, descriptive analyses were conducted using SPSS 26.0. Descriptive statistics for all study variables and Pearson’s correlations between them were calculated. Second, to reconfirm the longitudinal relationships between bullying victimization and T2 NSSI, regression analysis was conducted to determine the extent to which bullying victimization related to T2 NSSI. Third, to investigate the potential buffering effects of gratitude and parental autonomy support, moderation analyses were conducted using the process macro (Model 3) developed by Hayes (2021). In total, there were four interaction terms created: bullying victimization × gratitude, bullying victimization × parental autonomy support, parental autonomy support × gratitude, and bullying victimization × parental autonomy support × gratitude. When an interaction effect was significant, simple slope analyses (1 SD above and below the mean of the moderator) were conducted [72]. The significance of the longitudinal relationship at different levels of the moderator was tested using bias-corrected bootstrap confidence intervals (CIs) at 95% (5000 random samples). In steps 2 and 3, T1 NSSI and sociodemographic covariates (i.e., age, gender, and parental educational level) were statistically controlled for.

## 3. Results

### 3.1. Descriptive Statistics and Correlation Analyses

This research aimed to investigate how bullying victimization foreshadowed NSSI through moderating variables of gratitude and parental autonomy support. As shown in Table 1, bullying victimization was positively related to NSSI at both time points, whereas gratitude and parental autonomy support were negatively related to T1 and T2 NSSI. 

### 3.2. The Longitudinal Relationship between Bullying Victimization and T2 NSSI 

As presented in Table 2, bullying victimization was significantly related to T2 NSSI after controlling for T1 NSSI, age, gender, and parental educational level. This finding suggests that bullying victimization can have a long-term positive relationship with NSSI. 

### 3.3. Moderating Roles of Gratitude and Parental Autonomy Support

To test the moderation hypothesis, the relationships among bullying victimization, gratitude, parental autonomy support, and T2 NSSI were estimated using the PROCESS macro (Model 3) developed by Hayes (2021) [73]. The specifications of each model are summarized in Table 3. 

There were two significant interactions related to NSSI at T2: (1) a two-way interaction between parental autonomy support and bullying victimization (*b* = −0.12, *SE* = 0.05, *p* = 0.01); and (2) a three-way interaction among bullying victimization, parental autonomy support, and gratitude (*b* = 0.13, *SE* = 0.05, *p* = 0.01). No significant two-way interactions between gratitude and bullying victimization and between gratitude and parental autonomy support were observed. 

**Figure 1:**
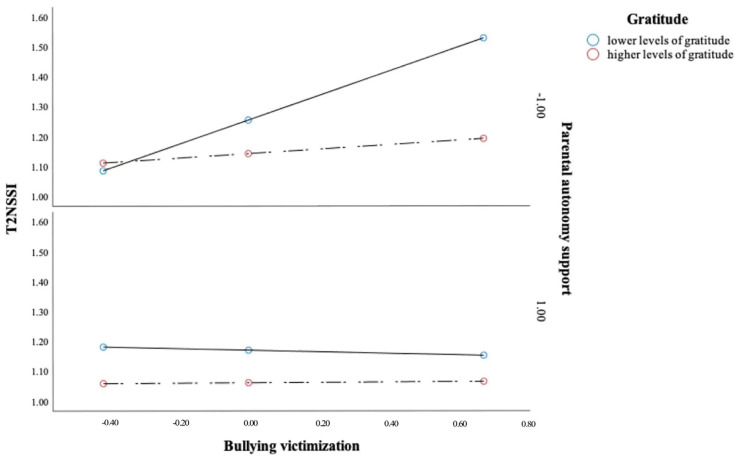
Interaction effect of bullying victimization, gratitude, and parental autonomy support on T2 NSSI. Parental autonomy support and gratitude were divided into two levels based on the mean: M − 1 SD and M + 1 SD. Note: Age, gender, parental educational level, and T1 NSSI were included as covariates of T2 NSSI.

As shown in Figure 1, decomposition of the three-way interaction using simple slope analysis revealed that, for adolescents with higher levels (one standard deviation below the sample mean) of gratitude, parental autonomy support did not moderate the link between bullying victimization and NSSI, such that bullying victimization was not related to T2 NSSI regardless of the levels of parental autonomy support (*b* = 0.07, *SE* = 0.04, *p* = 0.59 for M + 1 SD parental autonomy support; *b* = 0.01, *SE* = 0.07, *p* = 0.93 for M − 1 SD parental autonomy support). For adolescents with lower levels of gratitude, parental autonomy support did moderate the association between bullying victimization and NSSI, such that bullying victimization was significantly related to NSSI only when adolescents reported lower levels of parental autonomy support (*b* = 0.40, *SE* = 0.08, *p* < 0.001); in contrast, when adolescents reported experiencing higher levels of parental autonomy support, bullying victimization was unrelated to NSSI (*b* = −0.03, *SE* = 0.09, *p* = 0.78).

## 4. Discussion

The current study applied the socioecological theory [16] as an overarching framework with particular focuses on intrapersonal factors (i.e., gratitude) and interpersonal factors (i.e., parental autonomy support) in buffering the positive association between bullying victimization and NSSI through a two-wave longitudinal study. There were two major findings: (1) bullying victimization was positively related to NSSI one year later, even after controlling for the baseline level of NSSI; (2) such a positive association held true only for adolescents who experienced lower levels of gratitude and parental autonomy support. However, for adolescents who experienced higher gratitude, bullying victimization was not related to NSSI, regardless of the levels of parental autonomy support. The current study contributes to the extant research by investigating gratitude and parental autonomy support on the linkage between bullying victimization and adolescent NSSI. The findings collected in the present study contain important theoretical and practical implications, as we discussed below. 

### 4.1. The Linkage from Bullying Victimization to NSSI

As expected, the current study confirmed the positive associations between bullying victimization on NSSI both concurrently and longitudinally. Such a finding was in line with most of the previous studies [14,74], whereas it contradicted the findings reported by Heilbron and Prinstein (2010), showing that bullying victimization had no longitudinal impact on adolescent NSSI [15]. One possible interpretation for these inconsistent findings might be due to distinct covariates being included in those analyses. In accordance with Jiang et al. (2016)’s research, the current study statistically controlled for sociodemographic characteristics and baseline levels of NSSI [14]. In contrast, in Heilbron & Prinstein’s (2010) work, depressive symptoms were adjusted for [15]. Depression symptoms are a salient trigger for NSSI, perhaps leading to the fact that the unique effect of bully victimization on NSSI eclipses. Additionally, Heilbron & Prinstein (2010) followed their participants for two years [15]. This time interval between two-time points may be too long for victims to engage in NSSI. As stated by Jiang et al. (2016), bullying victimization may affect adolescent NSSI in a relatively shorter time period (e.g., one year) [14]. Taken together, the present findings support the interpersonal model of NSSI [19], indicating that bullying victimization is a negative interpersonal event causing NSSI. The present study also contributes to the existing literature by suggesting that bullying victimization has a longitudinal positive relationship on adolescent NSSI.

### 4.2. The Moderating Roles of Gratitude and Parental Autonomy Support

The key results of the current study revealed that for adolescents with higher levels of gratitude, parental autonomy support no longer plays a buffering role. That is when adolescents experience higher levels of gratitude, the incremental effects of bullying victimization and T2 NSSI were non-significant, regardless of the levels of parental autonomy support. In contrast, for adolescents with lower levels of gratitude, parental autonomy support did matter. Higher levels of parental autonomy support can successfully keep adolescents from T2 NSSI after bullying victimization; for adolescents whose parental autonomy support was insufficient, bullying victimization related to an increase in NSSI over a period of one year. Such findings were in line with prior studies documenting the protective role of parental autonomy support [75] as well as its joint effects with gratitude [32]. These findings suggest that gratitude and parental autonomy support can manifest in a compensatory interaction pattern to prevent NSSI for adolescent victims. In agreement with previous research [42,43,47], adolescents with lower gratitude usually perceive less social support, more negative emotions, and poor coping skills. In this context, adolescents with higher parental autonomy support take unfavorable conditions as a challenge, seek help from parents, and employ positive strategies to cope with the stress associated with victimization. Such findings highlight the view that possessing more protective resources (e.g., simultaneously experiencing gratitude and parental autonomy support) may help the individual to ward off the stress associated with bullying victimization, thereby decreasing the likelihood of NSSI. 

These findings have noteworthy theoretical and practical implications. Theoretically, first of all, the buffering roles of gratitude and parental autonomy support revealed in this research between bullying victimization and NSSI, suggesting the complexity of the occurrence of NSSI and enriching the socioecological theory [16]. Second, the moderating roles documented in the current study suggest a fruitful direction to solve the “puzzle” pertaining to the frequently found non-significant link between bullying victimization and NSSI [15]. Namely, it may be worthwhile to explore the other moderators that may hinder the process of bullying victimization to NSSI. 

Practically, the present study could be informative for school staff to wrestle with adolescent NSSI after bullying incidents. First, given that bullying victimization is a precipitating factor of NSSI, prevention and intervention programs should take into consideration anti-bullying policies and curriculum-based activities. Second, following the broaden-and-build theory [41], gratitude may contribute to positive emotions, thereby broadening their thought-action repertoires. Hence, fostering gratitude not only among victims of bullying but also among normative adolescents is a promising avenue for preventing victims from NSSI and enhancing adolescents’ overall well-being. There are three general methods of gratitude induction: gratitude lists, behavioral expressions of gratitude, and grateful contemplation [42]. Third, it is essential to forcefully inform parents about the positive effects of autonomy support in adolescent development so that even when their children experience victimization, parents would consistently render autonomy-supportive practices.

## 5. Limitations and Future Direction

The current study has some limitations, leaving exploration space for future research. First, all the measures in this study are self-reported. Although these measures were deliberately selected according to good psychometric properties, it is possible that the self-reported measures may be subject to respondent bias (i.e., social desirability). Future studies are suggested to collect data through multiple sources, such as the peer nomination method for bullying victimization. Second, the results were based on data from China. Culture may shape parents’ endorsement of autonomy support [70]. It is uncertain whether the findings would generalize to other cultures. Future studies that involve participants from other cultural groups are recommended to make a cross-cultural comparison. Third, the current study only investigated gratitude and parental autonomy support as intra-and interpersonal protective factors in alleviating the positive association between bullying victimization and NSSI. Other underexplored forms of positive intrapersonal (e.g., hope and resilience) and interpersonal factors (e.g., teacher autonomy support) need to be scrutinized in future initiatives. 

## 6. Conclusions

The present research sheds light on the longitudinal association between bullying victimization and NSSI and contributes to the scarce literature on the protecting roles of gratitude and parental autonomy support. The findings show that the longitudinal associations between bullying victimization and NSSI vary by the levels of gratitude or parental autonomy support. Specifically, gratitude and parental autonomy support, manifesting in a compensatory interaction pattern, serve as targeted agents for breaking the vicious linkage between bullying victimization and NSSI.

## Figures and Tables

**Table 1:** Means, standard deviations, and correlations among study variables.

Variables	1	2	3	4	5	6	7	8
1. Age	1							
2. Gender (male = 0, female = 1)	−0.08	1						
3. Parental educational level	0.03	−0.03	1					
4. Bullying victimization	0.02	−0.05	0.02	1				
5. T1 NSSI	−0.02	0.16 *	−0.08	0.43 ***	1			
6. T2 NSSI	−0.04	0.17 **	−0.07	0.29 ***	0.53 ***	1		
7. Gratitude	0.05	−0.11	0.14 *	−0.12	−0.32 ***	−0.32 ***	1	
8. Parental autonomy support	−0.04	−0.09	0.17 *	−0.09	−0.27 ***	−0.27 ***	0.41 ***	1
*M*	13.45	-	-	1.41	1.27	1.19	3.83	3.24
*SD*	0.53	-	-	0.66	0.71	0.53	0.73	1.02

Note: * *p* < 0.05, ** *p* < 0.01, *** *p* < 0.001.

**Table 2:** Regression analysis of the longitudinal association between T1 bullying victimization and T2 NSSI.

	*b*	*SE*	*t*	*p*	*R* ^2^	*F*
Bullying victimization	0.10	0.05	1.99	0.05	0.24	13.09
T1 NSSI	0.31	0.05	5.72	<0.001		
Age	−0.01	0.06	−0.27	0.86		
Gender	0.12	0.06	0.12	0.08		
Parental educational level	−0.01	0.03	−0.25	0.80		

**Table 3:** Regression analysis examining the associations of bullying victimization, gratitude, and parental autonomy support with NSSI.

Predictors	*b*	*SE*	*p*	95% CI	*R* ^2^	*F*
					0.37	10.25
T1 Bullying victimization	0.11	0.06	0.04	0.00~0.22		
T1 Gratitude	−0.08	0.05	0.10	−0.17~0.02		
T1 Parental autonomy support	−0.04	0.03	0.23	−0.11~0.03		
T1 Bullying victimization × T1 Gratitude	−0.11	0.08	0.17	−0.26~0.05		
T1 Bullying victimization × T1 Parental autonomy support	−0.12	0.05	0.01	−0.22~−0.03		
T1 Parental autonomy support × T1 Gratitude	0.001	0.04	0.98	−0.08~0.08		
T1 Bullying victimization × T1 Parental autonomy support × T1 Gratitude	0.13	0.05	0.01	0.03~0.23		
T1 NSSI	0.19	0.06	0.002	0.07~0.31		
T1 Age	−0.02	0.06	0.66	−0.13~0.09		
Gender	0.14	0.06	0.02	0.02~0.26		
Parental educational level	−0.02	0.03	0.58	−0.08~0.05		

## Data Availability

The data is available upon reasonable request from the first author.

## References

[1] Volk A.A., Dane A.V., Marini Z.A. (2014). What is bullying? A theoretical redefinition. Dev. Rev..

[2] Moore S.E., Norman R.E., Suetani S., Thomas H.J., Sly P.D., Scott J.G. (2017). Consequences of bullying victimization in childhood and adolescence: A systematic review and meta-analysis. World J. Psychiatry.

[3] Barzilay S., Brunstein Klomek A., Apter A., Carli V., Wasserman C., Hadlaczky G., Hoven C.W., Sarchiapone M., Balazs J., Kereszteny A. (2017). Bullying victimization and suicide ideation and behavior among adolescents in Europe: A 10-country study. J. Adolesc. Health.

[4] Pellegrini A.D., Long J.D. (2002). A longitudinal study of bullying, dominance, and victimization during the transition from primary school through secondary school. Br. J. Dev. Psychol..

[5] Haltigan J.D., Vaillancourt T. (2014). Joint trajectories of bullying and peer victimization across elementary and middle school and associations with symptoms of psychopathology. Dev. Psychol..

[6] Koyanagi A., Oh H., Carvalho A.F., Smith L., Haro J.M., Vancampfort D., Stubbs B., DeVylder J.E. (2019). Bullying victimization and suicide attempt among adolescents aged 12–15 years from 48 countries. J. Am. Acad. Child Adolesc. Psychiatry.

[7] Sawyer S.M., Azzopardi P.S., Wickremarathne D., Patton G.C. (2018). The age of adolescence. Lancet Child Adolesc..

[8] Kandel D.B. (1986). Processes of peer influences in adolescence. Development as Action in Context.

[9] Eastman M., Foshee V., Ennett S., Sotres-Alvarez D., Reyes HL M., Faris R., North K. (2018). Profiles of internalizing and externalizing symptoms associated with bullying victimization. J. Adolesc..

[10] Hay C., Meldrum R. (2010). Bullying victimization and adolescent self-harm: Testing hypotheses from general strain theory. J. Youth Adolesc..

[11] Van Geel M., Goemans A., Vedder P. (2015). A meta-analysis on the relation between peer victimization and adolescent non-suicidal self-injury. Psychiatry Res..

[12] Wu N., Hou Y., Zeng Q., Cai H., You J. (2021). Bullying experiences and nonsuicidal self-injury among Chinese adolescents: A longitudinal moderated mediation model. J. Youth Adolesc..

[13] Wang Q., Liu X. (2019). Peer victimization, depressive symptoms and non-suicidal self-injury behavior in Chinese migrant children: The roles of gender and stressful life events. Psychol. Res. Behav. Manag..

[14] Jiang Y., You J., Hou Y., Du C., Lin M.P., Zheng X., Ma C. (2016). Buffering the effects of peer victimization on adolescent non-suicidal self-injury: The role of self-compassion and family cohesion. J. Adolesc..

[15] Heilbron N., Prinstein M.J. (2010). Adolescent peer victimization, peer status, suicidal ideation, and nonsuicidal self-injury: Examining concurrent and longitudinal associations. Merrill. Palmer Q..

[16] Bronfenbrenner U. (1979). The Ecology of Human Development: Experiments by Nature and Design.

[17] Rutter M. (1985). Resilience in the face of adversity. Protective factors and resistance to psychiatric disorder. Br. J. Psychiatry.

[18] Nock M.K., Favazza A.R. (2009). Nonsuicidal self-injury: Definition and classification. Understanding Nonsuicidal Self-Injury: Origins, Assessment, and Treatment.

[19] Nock M.K. (2010). Self-injury. Annu. Rev. Clin. Psychol..

[20] Prinstein M.J., Guerry J.D., Browne C.B., Rancourt D. (2009). Interpersonal Models of Nonsuicidal Self-Injury. Understanding Nonsuicidal Self-Injury: Origins, Assessment, and Treatment.

[21] Nock M.K., Prinstein M.J. (2004). A functional approach to the assessment of self-mutilative behavior. J. Consult. Clin. Psychol..

[22] Coates A.A., Messman-Moore T.L. (2014). A structural model of mechanisms predicting depressive symptoms in women following childhood psychological maltreatment. Child Abuse Negl..

[23] Chu X.-W., Fan C.-Y., Liu Q.-Q., Zhou Z.-K. (2019). Rumination mediates and moderates the relationship between bullying victimization and depressive symptoms in Chinese early adolescents. Child Indic. Res..

[24] Wu X., Qi J., Zhen R. (2021). Bullying victimization and adolescents’ social anxiety: Roles of shame and self-esteem. Child Indic. Res..

[25] Gilbert P. (1997). The evolution of social attractiveness and its role in shame, humiliation, guilt and therapy. Br. J. Med. Psychol..

[26] Greene D.C., Britton P.J. (2013). The influence of forgiveness on lesbian, gay, bisexual, transgender, and questioning individuals’ shame and self-esteem. J. Couns. Devel..

[27] Gerard J.M., Booth M.Z. (2015). Family and school influences on adolescents’ adjustment: The moderating role of youth hopefulness and aspirations for the future. J. Adolesc..

[28] Extremera N., Quintana-Orts C., Merida-Lopez S., Rey L. (2018). Cyberbullying victimization, self-esteem and suicidal ideation in adolescence: Does emotional intelligence play a buffering role?. Front. Psychol..

[29] Rey L., Merida-Lopez S., Sanchez-Alvarez N., Extremera N. (2019). When and how do emotional intelligence and flourishing protect against suicide risk in adolescent bullying victims?. Int. J. Environ. Res. Public Health.

[30] Claes L., Luyckx K., Baetens I., Van de Ven M., Witteman C. (2015). Bullying and victimization, depressive mood, and non-suicidal self-injury in adolescents: The moderating role of parental support. J. Child Fam. Stud..

[31] Ledwell M., King V. (2015). Bullying and internalizing problems: Gender differences and the buffering role of parental communication. J. Fam. Issues.

[32] Ma C., Ma Y., Lan X. (2022). Parental autonomy support and pathological internet use among Chinese undergraduate students: Gratitude moderated the mediating effect of filial piety. Int. J. Environ. Res. Public Health.

[33] McCullough M.E., Emmons R.A., Tsang J.A. (2002). The grateful disposition: A conceptual and empirical topography. J. Pers. Soc. Psychol..

[34] Wood A.M., Maltby J., Stewart N., Joseph S. (2008). Conceptualizing gratitude and appreciation as a unitary personality trait. Pers. Individ. Differ..

[35] Wong Y.J., Owen J., Gabana N.T., Brown J.W., McInnis S., Toth P., Gilman L. (2018). Does gratitude writing improve the mental health of psychotherapy clients? Evidence from a randomized controlled trial. Psychother. Res..

[36] Kleiman E.M., Adams L.M., Kashdan T.B., Riskind J.H. (2013). Grateful individuals are not suicidal: Buffering risks associated with hopelessness and depressive symptoms. Pers. Individ. Differ..

[37] Mary E.M., Patra S. (2015). Relationship between forgiveness, gratitude and resilience among the adolescents. Indian J. Posit. Psychol..

[38] Williams L.A., Bartlett M.Y. (2015). Warm thanks: Gratitude expression facilitates social affiliation in new relationships via perceived warmth. Emotion.

[39] Lee J.-Y., Kim S.-Y., Bae K.-Y., Kim J.-M., Shin I.-S., Yoon J.-S., Kim S.-W. (2018). The association of gratitude with perceived stress and burnout among male firefighters in Korea. Pers. Individ. Differ..

[40] Hoy B.D., Suldo S.M., Mendez L.R. (2013). Links between parents’ and children’s levels of gratitude, life satisfaction, and hope. J. Happiness Stud..

[41] Fredrickson B.L. (2004). The broaden–and–build theory of positive emotions. Philos. Trans. R. Soc. B Biol. Sci..

[42] Wood A.M., Froh J.J., Geraghty A.W. (2010). Gratitude and well-being: A review and theoretical integration. Clin. Psychol. Rev..

[43] Petrocchi N., Couyoumdjian A. (2015). The impact of gratitude on depression and anxiety: The mediating role of criticizing, attacking, and reassuring the self. Self Identity.

[44] Bryan J.L., Young C.M., Lucas S., Quist M.C. (2018). Should I say thank you? Gratitude encourages cognitive reappraisal and buffers the negative impact of ambivalence over emotional expression on depression. Pers. Individ. Differ..

[45] Lo H.H.M., Kwok SY C.L., Yeung JW K., Low AY T., Tam CH L. (2017). The moderating effects of gratitude on the association between perceived parenting styles and suicidal ideation. J. Child Fam. Stud..

[46] Gross J.J. (1998). Antecedent-and response-focused emotion regulation: Divergent consequences for experience, expression, and physiology. J. Pers. Soc. Psychol..

[47] Kong F., Ding K., Zhao J. (2015). The relationships among gratitude, self-esteem, social support and life satisfaction among undergraduate students. J. Happiness Stud..

[48] Lin C.-C. (2016). The roles of social support and coping style in the relationship between gratitude and well-being. Pers. Individ. Differ..

[49] Disabato D.J., Kashdan T.B., Short J.L., Jarden A. (2017). What predicts positive life events that influence the course of depression? A longitudinal examination of gratitude and meaning in life. Cogn. Ther. Res..

[50] Nezlek J.B., Newman D.B., Thrash T.M. (2017). A daily diary study of relationships between feelings of gratitude and well-being. J. Positive Psychol..

[51] Gungor A., Young M.E., Sivo S.A. (2021). Negative life events and psychological distress and life satisfaction in US college students: The moderating effects of optimism, hope, and gratitude. J. Pedag. Res..

[52] Rey L., Quintana-Orts C., Merida-Lopez S., Extremera N. (2019). Being bullied at school: Gratitude as potential protective factor for suicide risk in adolescents. Front. Psychol..

[53] Brausch A.M., Gutierrez P.M. (2010). Differences in non-suicidal self-injury and suicide attempts in adolescents. J. Youth Adolesc..

[54] Scott L.N., Pilkonis P.A., Hipwell A.E., Keenan K., Stepp S.D. (2015). Non-suicidal self-injury and suicidal ideation as predictors of suicide attempts in adolescent girls: A multi-wave prospective study. Compr. Psychiatry.

[55] Klonsky E.D., May A.M., Saffer B.Y. (2016). Suicide, suicide attempts, and suicidal ideation. Annu. Rev. Clin. Psychol..

[56] Deci E.L., Ryan R.M. (2000). The “what” and “why” of goal pursuits: Human needs and the self-determination of behavior. Psychol. Inq..

[57] Feng L., Lan X. (2020). The moderating role of autonomy support profiles in the association between grit and externalizing problem behavior among family-bereaved adolescents. Front Psychol..

[58] Simon P.D. (2021). Parent autonomy support as moderator: Testing the expanded perfectionism social disconnection model. Pers. Individ. Differ..

[59] Weinstein N., Ryan R.M. (2011). A self-determination theory approach to understanding stress incursion and responses. Stress Health.

[60] Van der Giessen D., Branje S., Meeus W. (2014). Perceived autonomy support from parents and best friends: Longitudinal associations with adolescents’ depressive symptoms. Soc. Dev..

[61] Emery A.A. (2016). Applying Self-Determination Theory to Further Our Understanding of Non-Suicidal Self-Injury.

[62] Cohen S., Wills T.A. (1985). Stress, social support, and the buffering hypothesis. Psychol. Bull..

[63] Newman M.L., Holden G.W., Delville Y. (2005). Isolation and the stress of being bullied. J. Adolesc..

[64] Liew J., Kwok O., Chang Y.-P., Chang B.W., Yeh Y.-C. (2014). Parental autonomy support predicts academic achievement through emotion-related self-regulation and adaptive skills in Chinese American adolescents. Asian Am. J. Psychol..

[65] Wong M.M. (2008). Perceptions of parental involvement and autonomy support: Their relations with self-regulation, academic performance, substance use and resilience among adolescents. N. Am. J. Psychol..

[66] (2021). Foshan Bureau of Statistics. http://www.foshan.gov.cn/english/government/News/FoshanNews/content/post_5165416.html.

[67] Chang F.C., Lee C.M., Chiu C.H., Hsi W.Y., Huang T.F., Pan Y.C. (2013). Relationships among cyberbullying, school bullying, and mental health in Taiwanese adolescents. J. School Health.

[68] Kong F., Zhao J., You X., Xiang Y. (2020). Gratitude and the brain: Trait gratitude mediates the association between structural variations in the medial prefrontal cortex and life satisfaction. Emotion.

[69] Jiang Y., Ren Y., Zhu J., You J. (2020). Gratitude and hope relate to adolescent nonsuicidal self-injury: Mediation through self-compassion and family and school experiences. Curr. Psychol..

[70] Wang Q., Pomerantz E.M., Chen H. (2007). The role of parents’ control in early adolescents’ psychological functioning: A longitudinal investigation in the United States and China. Child Dev..

[71] Wang Q., Chan H.-W., Lin L. (2012). Antecedents of Chinese parents’ autonomy support and psychological control: The interplay between parents’ self-development socialization goals and adolescents’ school performance. J. Youth Adolesc..

[72] Aiken L.S., West S.G., Reno R.R. (1991). Multiple Regression: Testing and Interpreting Interactions.

[73] Hayes A.F. (2021). Introduction to Mediation, Moderation, and Conditional Process Analysis: A Regression-Based Approach.

[74] Baiden P., Stewart S.L., Fallon B. (2017). The mediating effect of depressive symptoms on the relationship between bullying victimization and non-suicidal self-injury among adolescents: Findings from community and inpatient mental health settings in Ontario, Canada. Psychiatry Res..

[75] Thomassin K., Suveg C. (2012). Parental autonomy support moderates the link between ADHD symptomatology and task perseverance. Child Psychiatry Hum. Dev..

